# Effects of drought pretreatment on the morphology traits, biomass, and stoichiometric characteristics of the desert ephemeral plant

**DOI:** 10.3389/fgene.2025.1534894

**Published:** 2025-03-20

**Authors:** Qian Liu, Hongmin Li, Chen Gong, Qianli Zhang, Tao Pan, Zhaodan Cao, Yanfeng Chen

**Affiliations:** School of Geography and Tourism, Qufu Normal University, Rizhao, Shandong, China

**Keywords:** drought pretreatment, overcompensation effect, *Erodium oxyrhinchum*, ephemeral plant, the Gurbantunggut desert

## Abstract

**Introduction:** In the context of climate change, the frequency and intensity of droughts in arid and semi-arid areas have shown a substantially increasing trend, which inevitably affects plant survival and growth. However, it is unclear what survival and growth strategies plants subjected to drought pretreatment in the early life stages adopt when facing subsequent drought stress.

**Methods:** Here, we conducted a field experiment and set up two treatments, control and drought pretreatment, to investigate the effects of drought pretreatment on the survival, phenology, morphology, biomass, and stoichiometric characteristics of the ephemeral plant *Erodium oxyrhinchum* in the Gurbantunggut Desert, China.

**Results:** The results showed that the leafing, flowering, and fruiting stages under drought pretreatment occurred markedly earlier than the control treatment by 5.25 ± 1.2 d, 3.13 ± 0.84 d, and 4.75 ± 1.63 d, respectively. The life history of *E. oxyrhinchum* decreased 5 ± 1.38 d under drought pretreatment. Drought pretreatment accelerated seedling mortality, leading to a faster and earlier decline in survival percentage. The survival percentage of *E. oxyrhinchum* under drought pretreatment at the full blooming stage was approximately 18.59%, which was 5.19% higher than that of the control treatment. In addition, a positive correlation was observed between morphological traits and individual biomass, and drought pretreatment substantially increased individual biomass and reproductive output. For example, the reproductive biomass under drought pretreatment was 1.41 times than that of the control treatment during the full fruiting stage, indicating that plants subjected to drought pretreatment exhibited an overcompensation effect. Finally, from the perspective of stoichiometric characteristics, plants subjected to drought pretreatment require more phosphorus to enhance their resistance to severe drought.

**Conclusion:** This study provides novel insights for the conservation and restoration of desert ecosystems in the context of climate change.

## 1 Introduction

Drought is expected to increase in frequency, severity, and duration in the future because of global warming, which will pose serious threats to plant survival, growth, and regeneration (Lin et al., 2014; Huang et al., 2015a). Many studies have indicated that drought can affect multiple life history stages of plants, such as by decreasing germination percentage, increasing seedling mortality, and altering biomass allocation (Zia et al., 2021; Walters et al., 2023; Zhang et al., 2023; Brunet, et al., 2024). However, some researchers have found that plants can adopt various strategies to cope with drought exposure, including changes in root configuration, reduced leaf area, regulation of antioxidant accumulation, and ratios of C, N, and P (Guo et al., 2023; Fuente et al., 2023; Haghpanah et al., 2024). In particular, when plants are rewatered after an initial drought, they often show an overcompensation effect and improve their adaptability to drought stress (Hofer et al., 2017; Gao et al., 2024). Therefore, it is crucial to investigate the effects of drought pretreatment on plants in the context of global climate change.

Plants subjected to drought pretreatment may exhibit coping strategies compared to those that have experienced a single drought event (Haider et al., 2024). Consequently, numerous scholars have explored the effects of drought pretreatment on plants from multiple perspectives, including morphological changes, biomass allocation, and stoichiometry composition. For example, Gao et al. (2024) found that the root length of maize seedlings increased by 21.05% after drought rewatering, enhancing the ability of maize seedlings to adapt to drought stress following two cycles of drought rewatering treatments. Lv et al. (2024) also found that drought pretreatment not only increased the number of small vascular bundles, altered the shape and density of stomata, and enhanced water use efficiency in potatoes, but also increased the amount and rate of dry biomass transport, thereby reducing the adverse effects of drought stress on potato tubers. Another study from the perspective of the content and ratios of stoichiometry also found that the content of N in different organs and P in the fine roots of *Pinus yunnanensis* seedlings increased after rewatering with light drought, moderate drought, and severe drought treatments, while the C:N ratio decreased (Liu et al., 2024). These findings indicate that drought pretreatment can increase the root length of maize seedlings and the dry biomass of potatoes, altering the allocation of stoichiometric composition through an overcompensated effect. However, most studies have focused on crops or tree species, with relatively little research on the effects of drought pretreatment on desert herbs in arid environment.

The frequency and severity of drought events are increasingly influenced by global climate change, particularly in arid and semiarid regions (Ghannoum, 2009; Hosseinizadeh et al., 2015). In the temperate desert ecosystems of Central Asia, ephemeral plants play crucial roles in windbreaks and sand fixation, stabilising moving sand dunes, and protecting agricultural lands from sand encroachment (Sun et al., 2008; Mu et al., 2021; Lu et al., 2022). Moreover, these plants primarily rely on snowmelt and rainfall in early spring for rapid growth, completing their life history before the onset of hot and dry summers, and characterising them as typical drought-avoidant species (Mu et al., 2021). Consequently, ephemeral plants are considered ideal materials for studying plant responses to drought stress (Chen et al., 2019a; Mu et al., 2021). In particular, *E. oxyrhinchum* is the dominant ephemeral plant in the Gurbantunggut Desert (Mu et al., 2021). It germinates in March-April, flowers in April-May, and fruits in May-June, completing its life history in 2–3 months (Zhang and Chen, 2002; Wang and Zhang, 2010; Zhang et al., 2022a; Zhang et al., 2022b). This life strategy of *E. oxyrhinchum* enables it to evade extreme summer temperatures in arid regions. During the growing season of *E. oxyrhinchum*, soil moisture from snowmelt and spring rainfall exhibited a fluctuating downward trend, with soil dryness gradually increasing (Chen et al., 2019a). Studies have indicated that the soil water content in early spring typically remains above 10%, and by the end of its life history, it can drop to less than 4% (Chen et al., 2019a). Other studies have shown that plants often increase the allocation of belowground biomass and alter their leaf morphology, transitioning from deeply lobed to entirely palmate leaves under drought conditions (Chen et al., 2019b). Based on the drought-escaping characteristics of ephemeral plants and the dynamic trends of soil water content in the Gurbantunggut Desert, we propose the first hypothesis: *E. oxyrhinchum* reduces its survival percentage, enhances drought tolerance, and triggers compensatory effects on morphology and biomass accumulation.

Furthermore, Carbon (C), nitrogen (N), and phosphorus (P) are essential for plant growth and physiological activities (Lu et al., 2023). Plants adapt to environmental changes through coordination among the elements in their bodies, which reflects the relationship between plants and their environment (Jiang et al., 2023). Accordingly, we proposed the second hypothesis: *E. oxyrhinchum* subjected to drought pretreatment will exhibit significant increases in the concentration of C, N, and P in leaves. To validate these hypotheses, we conducted a pre-drought experiment in the Gurbantunggut Desert to assess the effect of drought pretreatment on survival, phenology, morphology, biomass, and stoichiometric characteristics of *E. oxyrhinchum*. Therefore, this study provides a theoretical and practical basis for the adaptive evolution and conservation of ephemeral plants in desert environments.

## 2 Materials and methods

### 2.1 Study area

The study area is located on the southern edge of the Gurbantunggut Desert in Northwest China. The Gurbantunggut Desert, characterised by a typical temperate continental arid climate, is the largest stabilised and semi-stabilised desert in China (Chen et al., 2019c; Lu et al., 2022; Zhang et al., 2022b). The mean annual temperature varies from 6°C to 10°C, with extreme high temperatures over 40°C (Liu et al., 2020a). The annual potential evaporation can exceed 2,000 mm, and the annual precipitation in the central region of the desert is only 70–100 mm, which mainly occurs in winter and spring (Liu et al., 2020a; Lu et al., 2022). Natural conditions in the Gurbantunggut Desert are extremely harsh and susceptible to human activity and extreme climatic events. However, compared with other desert regions in China, herbaceous plants in the Gurbantunggut Desert are relatively abundant in spring, especially in ephemeral plant communities (Erfan et al., 2023). *E. oxyrhinchum* is a typical annual ephemeral plant found in the Gurbantunggut Desert. It contributes to more than 50% of the aboveground productivity of ephemeral plants, is the dominant species in the Gurbantunggut Desert, and plays a crucial role in the desert ecosystem of China (Zang et al., 2020).

### 2.2 Research methods

#### 2.2.1 Drought pretreatment and field data collection

**FIGURE 1:**
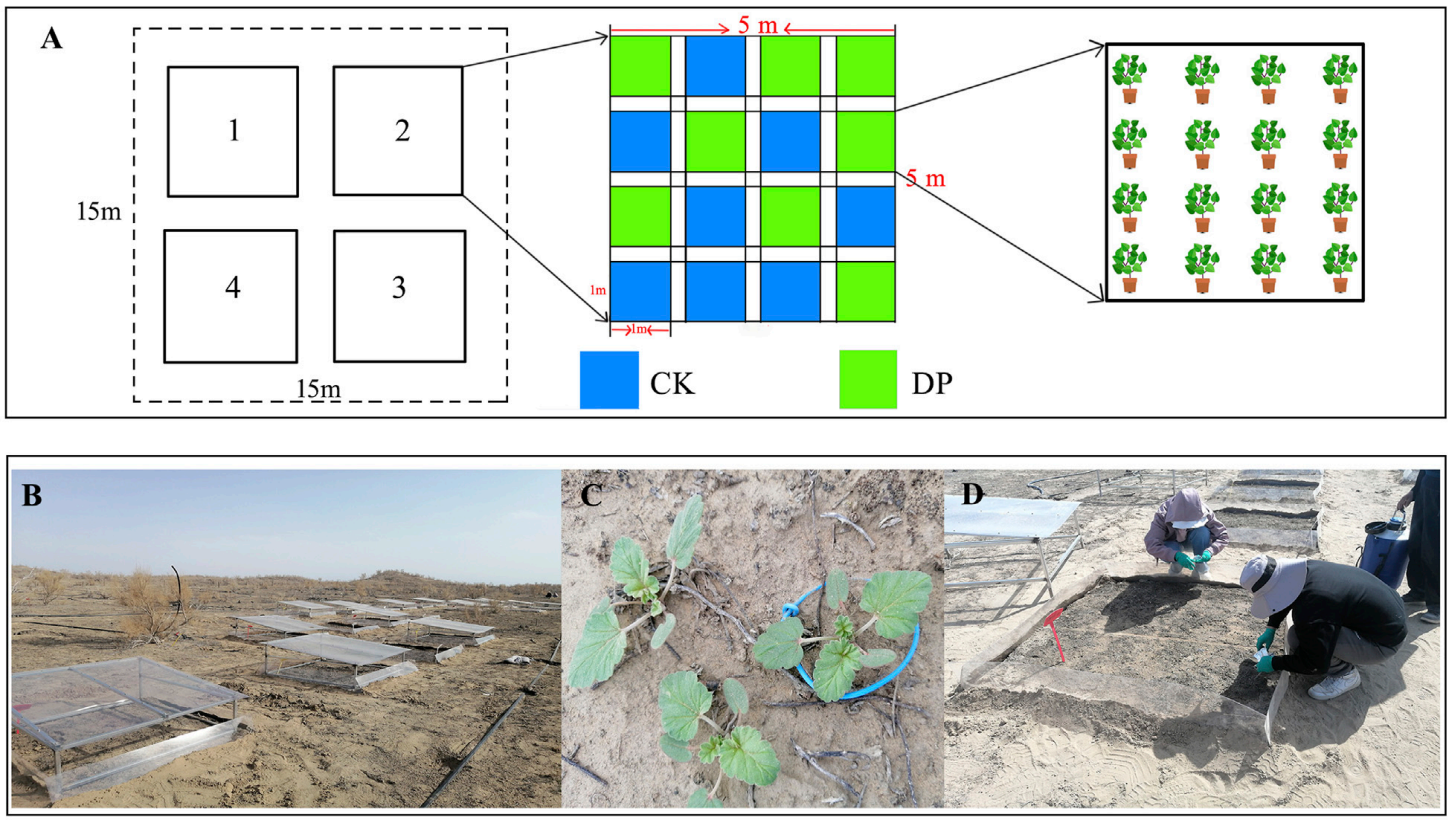
Experimental design **(A)**, PVC light-transmitting and rain-sheltering equipment **(B)**, *Erodium oxyrhinchum* seedlings **(C)** and sample collection **(D)** in the field. Note: Control treatment, CK; Drought pretreatment, DP.

The field experimental plot is located at the southern edge of the Gurbantunggut Desert (44°26′N, 87°54′E), which was established in September 2015. Firstly, in order to prevent interference from wildlife and humans, we have established reinforced bilateral fences. Secondly, we have installed PVC light-transmitting and rain-sheltering equipment, it can be used to block the interference of natural precipitation. Finally, a 15 m × 15 m plot with relatively flat terrain and no distribution of *E. oxyrhinchum* was selected within the aforementioned field plot. A randomized block design was used, with four large plots (5 m × 5 m) spaced 3 m apart. Within each large plot, 16 small plots (1 m × 1 m) were set up, with 8 plots designated for control treatment and 8 plots for drought pretreatment (Figures 1A, B). A 5 mm thick water-impermeable membrane was used between the large and small plots to prevent water seepage.

**FIGURE 2:**
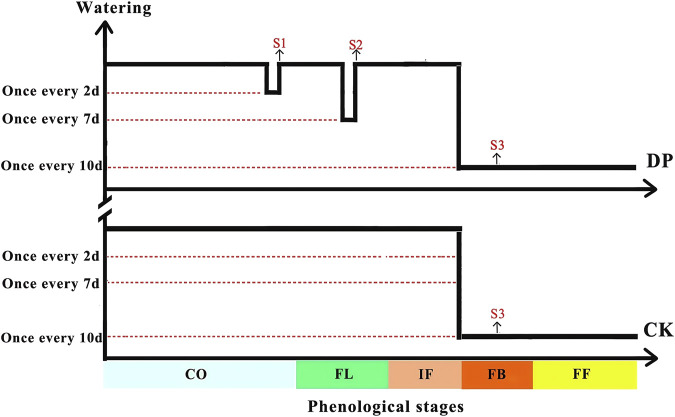
Experimental design of drought pretreatment for the desert ephemeral plant *Erodium oxyrhinchum*. Note: Control treatment, CK; Drought pretreatment, DP; Cotyledon stage, CO; Full leaf expansion stage, FL; Initial flowering stage, IF; Full blooming stage, FB; Full fruiting stage, FF; Mild drought, S1; Moderate drought, S2; Severe drought, S3.

Considering the average precipitation (102 mm) during the growth season of ephemeral plants in the Gurbantunggut desert over the past decade, we conducted an experiment of drought pretreatment by controlling watering frequency on the basis of complete rain cover. For the control treatment (CK): watering once every 2 days during the cotyledon, leaf expansion, and initial flowering stages, and once every 10 days during the full booming, fruiting and withering stages, with each watering amount of 8 mm (calculated based on the average watering amount of the life history and phenological transition time of *E. oxyrhinchum*). The cumulative watering volume for the control treatment was approximately 104 mm. For the drought pretreatment (DP): watering once every 4 days during the cotyledon stage to simulate mild drought, watering once every 7 days during the full leaf expansion stage to simulate moderate drought, and once every 10 days during the full blooming, fruiting and withering stages to simulate severe drought (Figure 2). And once every 2 days during other phenological stages consistent with the control treatment (Figure 2). The cumulative watering amount was about 56 mm.

Plant samples were collected at different phenological stages of *E. oxyrhinchum*: the cotyledon stage (29 March), initial leaf expansion stage (mid-April), full leaf expansion stage (late April), initial flowering stage (early May), full booming stage (mid-May), and full fruiting stage (late May). Whole plants were preserved in water bags and transported to the laboratory for analysis, and the leaves of *E. oxyrhinchum* were preserved in liquid nitrogen to determine the stoichiometric indicators (Figures 1C, D).

#### 2.2.2 Plant phenology investigation and measurement of morphology, biomass, and stoichiometric indicators

##### 2.2.2.1 Phenology survey

For each treatment, the number of days from 1 January 2023 to the germination, leafing, blooming, fruiting, and withering stages was calculated. These data served as the phenological metrics for subsequent analyses.

##### 2.2.2.2 Morphology and biomass traits

As the plants grew, their traits (plant height, leaf area, root length, and number of leaves) were measured. We measured the leaf area of fresh leaves using an LI-COR 3000 leaf area meter. After the plant traits were measured, the plants in all treatments were harvested and separated into roots, stems, leaves, and reproductive organs (flowers, fruits, and seeds). The roots were carefully washed free of soil. Plant samples were blanched in an oven at 105°C for 30 min before drying at 70°C to a consistent weight. The plant organs were weighed using an electronic balance (OHAUS, Pine Brook, NJ, United States, 0.0001 g). Individual biomass (IB) was determined by adding the aboveground biomass (AGB) and belowground biomass (BGB), and the root to shoot ratio (R/S) was computed as the ratio of belowground to aboveground biomass.

##### 2.2.2.3 Stoichiometric measurement

Mature leaves from the upper section of *E. oxyrhinchum* were dried and ground into powder using a ball mill for stoichiometric analysis. The C and N concentrations in the leaves were determined using an elemental analyser (EA3000, Italy), and the P concentration was determined using the molybdenum-antimony colorimetric method.

### 2.3 Statistical analysis

The survival percentage of *E. oxyrhinchum* was analysed using independent sample t-tests with a significance level of 0.05, and Levene’s test was used to confirm the homogeneity of variance. In addition, independent sample t-tests (consistent with the survival percentage analysis method) and two-way ANOVA were used to analyse plant height, root length, number of leaves, leaf area, individual biomass, reproductive biomass, root to shoot ratio, growth rate, and stoichiometric characteristics. The coefficient of variation (CV) was employed to examine the variability of leaf C, N, and P concentration and their ratios throughout the sampling period: 0≤CV<15% shows weak variability, 15%≤CV<35% indicates moderate variability, and CV ≥ 35% indicates high variability. Data analysis was conducted using SPSS27.0, and graphical representations were generated using R-4.3.2.

## 3 Results

### 3.1 The effects of drought pretreatment on the phenology and survival percentage of *E. oxyrhinchum*

Statistical analysis revealed that the phenological stages of *E. oxyrhinchum* under drought pretreatment were significantly earlier than those under the control treatment (*p* < 0.001, Table 1). Specifically, under drought pretreatment, the full leaf expansion stage occurred 5.25 ± 1.2 days earlier, the initial flowering stage 3.13 ± 0.84 days earlier, the initial fruiting stage 4.75 ± 1.63 days earlier, and the withering stage 5 ± 1.38 days earlier than that of control treatment (Table 1). Notably, the entire life history of *E. oxyrhinchum* under drought pretreatment were also significant shorter than that of control treatment: DP (69.63 ± 0.75 days) < CK (74.63 ± 0.63 days).

**TABLE 1:** Effect of drought pretreatment on phenological stages of *Erodium oxyrhinchum*.

Phenological stages	CK	SD	DP	SD
Cotyledon stage	78.00 days	0.00	78.00 days	0.00
Initial leaf expansion stage	88.00 days	0.00	88.00 days	0.00
Full leaf expansion stage	112.25 days	0.70	107.00 days	0.50
Initial flowering stage	124.63 days	0.38	121.50 days	0.46
Full blooming stage	131.50 days	0.63	128.63 days	0.60
Initial fruiting stage	132.13 days	0.74	127.38 days	0.89
Full fruiting stage	143.00 days	0.96	139.25 days	1.03
Withering stage	152.63 days	0.63	147.63 days	0.75
Life history	74.63 days	0.63	69.63 days	0.75

Note: Control treatment, CK; drought pretreatment, DP.

**FIGURE 3:**
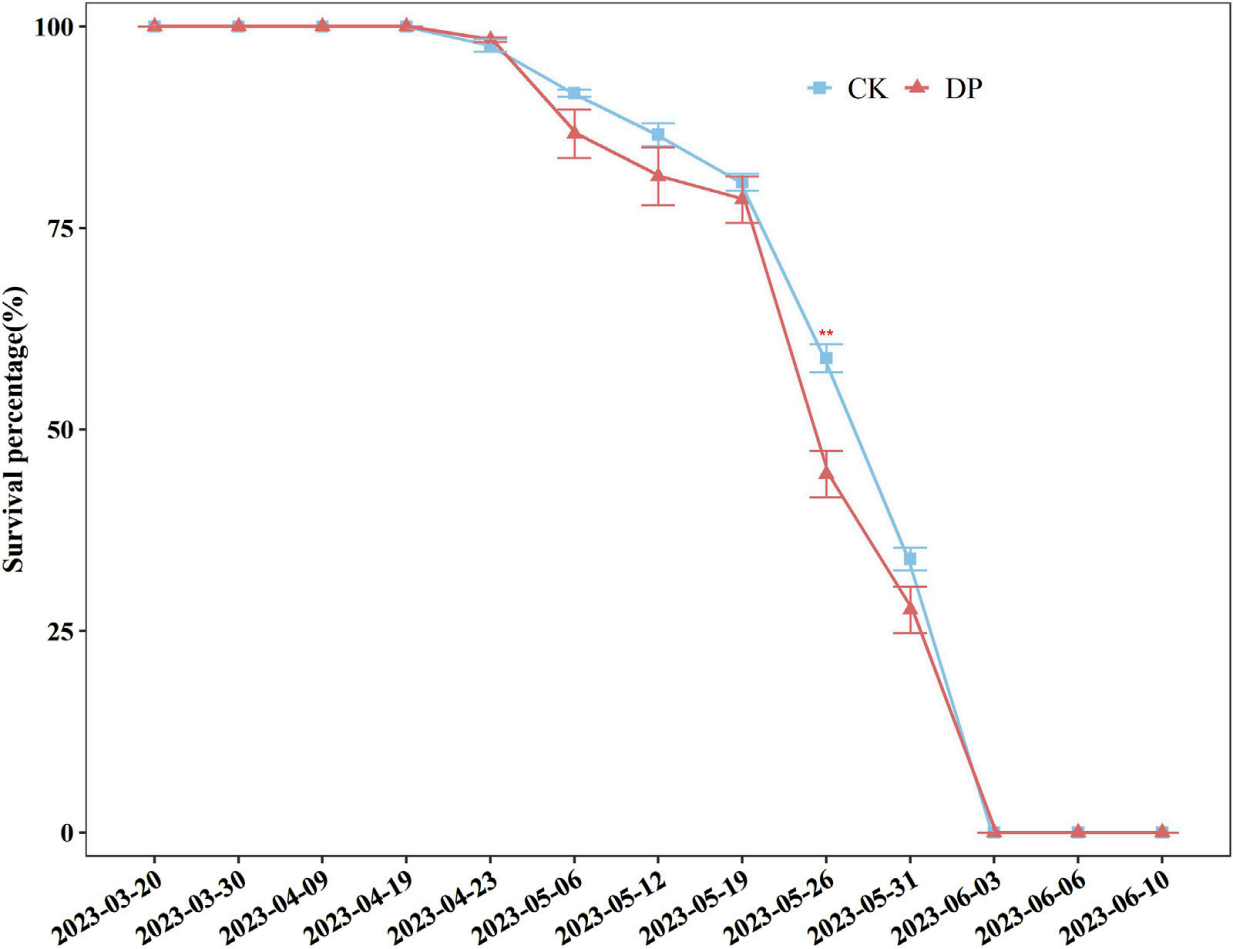
Effects of drought pretreatment on the survival percentage of *Erodium oxyrhinchum*. Note: Control treatment, CK; Drought pretreatment, DP; *p < 0.05, **p < 0.01, ***p < 0.001.

On 20 March 2023 the seeds of *E. oxyrhinchum* exhibited an explosive germination strategy, with seedlings appearing uniform before entering the cotyledon stage (Figure 3). The survival percentages of *E. oxyrhinchum* in the control and drought treatments did not significantly decrease from the cotyledon stage to April 19. After the full leaf expansion stage, the survival percentage of *E. oxyrhinchum* under drought pretreatment showed an earlier and faster decline than that of the control treatment (Figure 3). By the end of May, plants exposed to the drought pretreatment rapidly entered the withering stage. By early June, *E. oxyrhinchum* had completed its reproductive output and ended its life history between the control treatment and drought pretreatment (Figure 3).

### 3.2 The effects of drought pretreatment on the morphological traits of *E. oxyrhinchum*

The results of the two-way ANOVA indicated significant differences in terms of plant height, root length, leaf area, and leaf number of *E. oxyrhinchum* at different phenological stages (*p* < 0.001, Table 2). There were significant differences between the control and drought treatments in terms of plant height and root length (*p* < 0.05, Table 2); however, no significant differences were observed in leaf number (*p* = 0.502, Table 2) or leaf area (*p* = 0.12, Table 2). Furthermore, there was a significant interaction effect between drought treatment and phenological stage on plant height and leaf number (*p* < 0.05, Table 2).

**TABLE 2:** Summary of a two-way ANOVA showing the effects of different drought treatments and phenological stages on the morphology, biomass and stoichiometric characteristics of *Erodium oxyrhinchum*.

Index	Factor	df	F	P
Plant height	Treatment	1	49.769	<0.001
	Phenological stage	5	819.905	<0.001
	Treatment * Phenological stage	5	7.065	<0.001
Root length	Treatment	1	13.078	<0.001
	Phenological stage	5	240.614	<0.001
	Treatment * Phenological stage	5	1.301	0.269
Number of leaves	Treatment	1	0.454	0.502
	Phenological stage	5	70.933	<0.001
	Treatment * Phenological stage	5	2.590	0.030
Leaf area	Treatment	1	2.450	0.120
	Phenological stage	5	71.862	<0.001
	Treatment * Phenological stage	5	1.289	0.274
Individual biomass	Treatment	1	6.459	0.012
	Phenological stage	5	127.398	<0.001
	Treatment * Phenological stage	5	2.149	0.065
R/S	Treatment	1	7.607	0.007
	Phenological stage	5	106.120	<0.001
	Treatment * Phenological stage	5	1.146	0.341
Reproductive biomass	Treatment	1	13.422	0.001
	Phenological stage	2	186.096	<0.001
	Treatment * Phenological stage	2	4.053	0.023
Growth rate	Treatment	4	146.208	<0.001
	Phenological stage	1	2.024	0.158
	Treatment * Phenological stage	4	0.680	0.608
C	Treatment	1	17.880	<0.001
	Phenological stage	5	20.665	<0.001
	Treatment * Phenological stage	5	12.749	<0.001
N	Treatment	1	83.056	<0.001
	Phenological stage	5	23.896	<0.001
	Treatment * Phenological stage	5	43.957	<0.001
P	Treatment	1	115.404	<0.001
	Phenological stage	5	75.809	<0.001
	Treatment * Phenological stage	5	18.665	<0.001
C:N	Treatment	1	12.393	0.002
	Phenological stage	5	55.059	<0.001
	Treatment * Phenological stage	5	3.313	0.020
C:P	Treatment	1	23.876	<0.001
	Phenological stage	5	47.162	<0.001
	Treatment * Phenological stage	5	3.028	0.029
N:P	Treatment	1	13.951	0.002
	Phenological stage	5	443.606	<0.001
	Treatment * Phenological stage	5	30.558	<0.001

**FIGURE 4:**
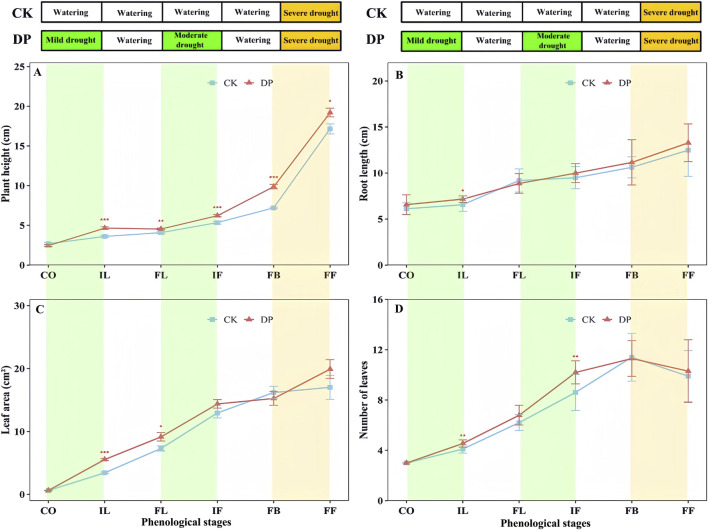
Effects of drought pretreatment on the plant height **(A)**, root length **(B)**, leaf area **(C)**, and number of leaves **(D)** of *Erodium oxyrhinchum*. Note: Control treatment, CK; Drought pretreatment, DP; Cotyledon stage, CO; Initial leaf expansion stage, IL; Full leaf expansion stage, FL; Initial flowering stage, IF; Full blooming stage, FB; Full fruiting stage, FF; *p < 0.05, **p < 0.01, ***p < 0.001.

The height, root length, and leaf area of *E. oxyrhinchum* in the control and drought treatments increased gradually with the phenological stage (Figure 4). Prior to the fruiting stage, the number of leaves increases. From the initial leaf expansion stage to the full fruiting stage, plant height under drought pretreatment was significantly higher than that of the control treatment (*p* < 0.05, Figure 4). During the initial leaf expansion stage, root length under drought pretreatment was significantly longer than that of the control treatment. At the initial leaf expansion and flowering stages, the number of leaves under the drought pretreatment was significantly higher than that of the control treatment, but the difference did not reach a significant level during the full blooming and fruiting stages.

### 3.3 The effects of drought pretreatment on biomass accumulation and allocation of *Erodium oxyrhinchum*

**FIGURE 5:**
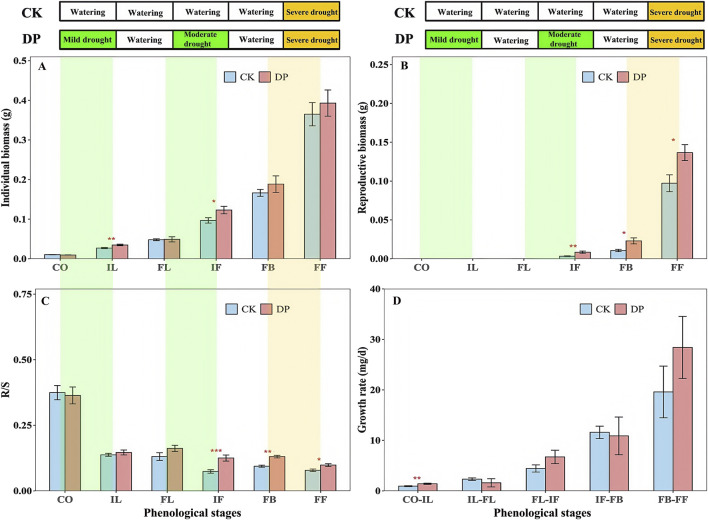
Effects of drought pretreatment on the individual biomass (**A**), reproductive biomass (**B**), R/S (**C**), growth rate (**D**) of *Erodium oxyrhinchum*. Note: Control treatment, CK; Drought pretreatment, DP; Cotyledon stage, CO; Initial leaf expansion stage, IL; Full leaf expansion stage, FL; Initial flowering stage, IF; Full blooming stage, FB; Full fruiting stage, FF; *p < 0.05, **p < 0.01, ***p < 0.001.

According to the results of the two-way ANOVA, the individual biomass, reproductive biomass, and growth rate of *E. oxyrhinchum* showed significantly increasing trends, but the R/S exhibited a significantly decreasing trend as the plants grew (*p* < 0.001, Table 2). Similarly, there were significant differences in the individual biomass, root to shoot ratio, and reproductive biomass of *E. oxyrhinchum* between the control and drought treatments (*p* < 0.05, Table 2). Throughout the reproductive season, the reproductive biomass under the drought pretreatment was consistently higher than that under the control treatment (Figure 5). Specifically, the reproductive biomass under drought pretreatment during the full fruiting stage was 1.41 times than that of the control. From the perspective of the entire life history, the individual biomass of *E. oxyrhinchum* under the drought pretreatment was greater than that of the control treatment (Figure 5). For example, during initial leaf expansion, the individual biomass of *E. oxyrhinchum* under the drought treatment was 1.29 times than that of the control treatment.

### 3.4 The effects of drought pretreatment on the C, N, and P stoichiometry characteristics of *E. oxyrhinchum*

**FIGURE 6:**
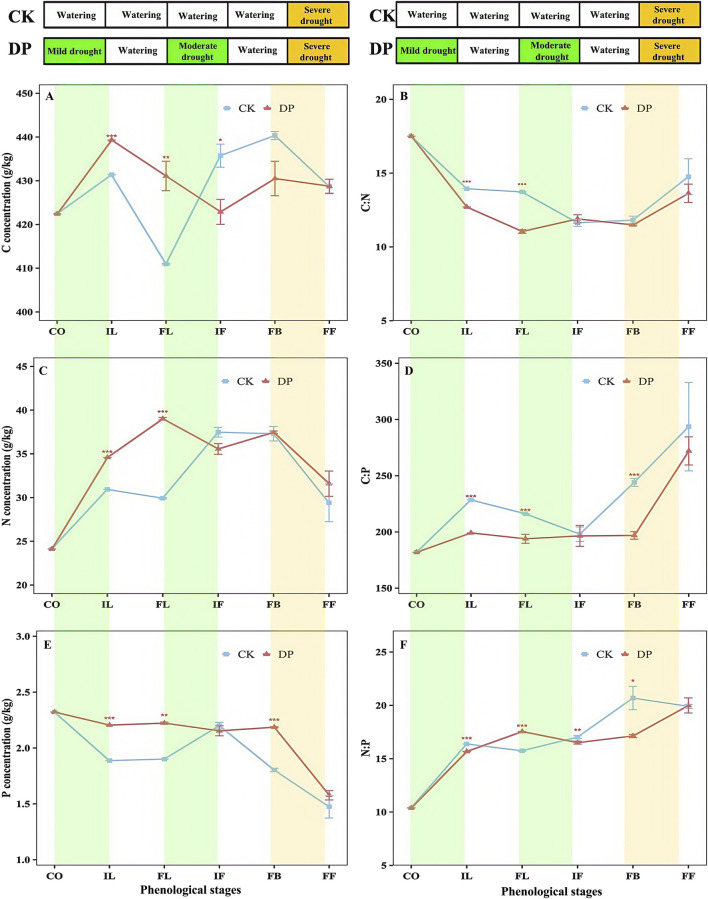
Effects of drought pretreatment on the C, N, and P stoichiometric characteristics of *Erodium oxyrhinchum*. Note: C concentration **(A)**; C:N ratio **(B)**; N concentration **(C)**; C:P ratio **(D)**; P concentration **(E)**; N:P ratio **(F)**; Control treatment, CK; Drought pretreatment, DP; Cotyledon stage, CO; Initial leaf expansion stage, IL; Full leaf expansion stage, FL; Initial flowering stage, IF; Full blooming stage, FB; Full fruiting stage, FF; *p < 0.05, **p < 0.01, ***p < 0.001.

Two-way ANOVA revealed significant differences in the stoichiometric characteristics and ratios of C: N, and P in the leaves of *E. oxyrhinchum* between the control and drought treatments (*p* < 0.001, Table 2). Similarly, from the perspective of the entire life history, significant differences in the stoichiometric characteristics and C: N, and P in the leaves of *E. oxyrhinchum* were observed at different phenological stages (*p* < 0.01). From the initial leaf expansion stage to the full leaf expansion stage, the C concentration in the leaves of *E. oxyrhinchum* under drought pretreatment was significantly higher than that in the control treatment (Figure 6A). However, at the initial flowering and full blooming stages, the C concentration under drought pretreatment was significantly lower than that of the control treatment. In addition, the N concentration in the leaves of *E. oxyrhinchum* exhibited a trend of initially increased and then decreased (Figure 6C). Before the initial flowering stage, the N concentration in the leaves of *E. oxyrhinchum* under the drought pretreatment was significantly higher than that in the control treatment. Similarly, from the perspective of the entire life history, the P concentration in the leaves of *E. oxyrhinchum* under drought pretreatment was significantly higher than that in the control treatment (Figure 6E).

The C: N ratio in the leaves of *E. oxyrhinchum* initially decreased and then increased as the phenological stage progressed, whereas the C:P and N:P exhibited an increasing trend. From the cotyledon stage to the full leaf expansion stage, the C: N in the leaves of *E. oxyrhinchum* under drought pretreatment was significantly lower than that in the control treatment (*p* < 0.001, Figure 6B). From the perspective of the entire life history, the C:P in the leaves of *E. oxyrhinchum* under drought pretreatment was also significantly lower than that in the control treatment (Figure 6D). Similarly, from the cotyledon stage to the full blooming stage, the N:P under the drought pretreatment was significantly lower than that under the control treatment (*p* < 0.05, Figure 6F).

From the perspective of the entire life history, the N and P concentration in the leaves of *E. oxyrhinchum* exhibited greater variation than the C concentration. Specifically, the coefficient of variation for C concentration in the leaves of *E. oxyrhinchum* between the control treatment and drought pretreatment was less than 5%, indicating weak variation (Table 3). However, from the full blooming stage to the full fruiting stage, the coefficient of variation for the N concentration under the control treatment and the P concentration under the drought pretreatment was greater than 15%, indicating moderate variation.

**TABLE 3:** Coefficient of variation of C, N, and P stoichiometric characteristics of *Erodium oxyrhinchum* at different phenological stages.

Index	Treatment	CO-IL	IL-FL	FL-IF	IF-FB	FB-FF
C	CK	1.15	2.66	3.28	0.91	1.55
	DP	2.15	1.34	1.54	1.59	1.11
N	CK	13.53	1.83	12.39	2.94	15.03
	DP	19.50	6.61	5.40	3.43	10.41
P	CK	11.31	0.40	8.16	11.01	12.95
	DP	2.82	0.63	2.85	2.40	17.88
C:N	CK	12.40	0.89	9.28	3.48	15.92
	DP	17.42	7.71	5.01	3.31	10.82
C:P	CK	12.44	3.05	5.16	11.59	13.72
	DP	4.97	1.95	3.37	3.19	17.91
N:P	CK	24.52	2.20	4.33	11.26	4.04
	DP	22.22	6.16	3.38	2.18	8.80

Note: Control treatment, CK; drought pretreatment, DP; cotyledon stage, CO; initial leaf expansion stage, IL; full leaf expansion stage, FL; initial flowering stage, IF; full blooming stage, FB; full fruiting stage, FF.

## 4 Discussion

This study aimed to investigate the effects of drought pretreatment on the survival, phenology, morphology, biomass, and stoichiometric characteristics of *E. oxyrhinchum* in the Gurbantunggut Desert. The results indicated that *E. oxyrhinchum* exposed to drought pretreatment exhibited significantly earlier phenological stages and lower survival percentages, and the reproductive biomass and individual biomass were significantly greater than those of the control treatment, partially supporting the first hypothesis. Furthermore, plants exposed to drought pretreatment needed to absorb more phosphorus than those exposed to the control treatment, but the differences in C and N in leaves did not reach a significant level, thus partially confirming the second hypothesis.

### 4.1 Effects of drought pretreatment on the phenology and survival percentage of *E. oxyrhinchum*

Phenology represents cyclical changes in vegetation and is a sensitive biological indicator of climate change (Li et al., 2021). This study revealed that the phenological stages of *E. oxyrhinchum* subjected to drought pretreatment were markedly accelerated. For instance, the full leaf expansion stage under drought pretreatment was 5.25 ± 1.2 days earlier than that of the control treatment. Early leafing allows plants to initiate photosynthesis sooner, thereby enhancing biomass accumulation (Ganjurjav et al., 2021; Amini et al., 2023; Wu and Yang, 2024). Similarly, the onset of flowering and fruiting of *E. oxyrhinchum* under drought pretreatment also occurred significantly earlier than in the control treatment. This may be because earlier leafing under drought pretreatment induced plants to enter the reproductive stage. Previous studies also have indicated that desert ephemeral plants employ a “drought escape strategy” by accelerating phenological stages to complete life history (Franks, 2011; Ding et al., 2022). These findings not only highlight the positive response of ephemeral plants to drought stress but also demonstrate that drought pretreatment can accelerate the rapid transitions of phenological stages, thereby influencing the life history of ephemeral plants.

The survival percentage reflects the adaptability of plants to environmental stress and is a valuable indicator of drought resistance in seedlings (He et al., 2024; Chen et al., 2025). In this study, the survival percentage of *E. oxyrhinchum* from March 20 to April 19 did not show a significant decrease trend, but the survival percentage of *E. oxyrhinchum* significantly decline after the full leaf expansion stage. The survival percentage of *E. oxyrhinchum* under drought pretreatment exhibited a faster and earlier declining trend than that of the control treatment. With the rapid growth of *E. oxyrhinchum*, the difference in survival percentage may be related to the decrease in soil water content and intensification of intraspecific competition. Similarly, Guo et al. (2020) investigated the combined effects of drought and intraspecific competition on the growth of *C. lanceolata*, and the results indicated that intense competition imposed by neighbours was a great threat to the survival of young *C. lanceolata* plants under prolonged drought. Furthermore, the trend in soil water content revealed lower moisture levels under drought pretreatment (Supplementary Figure 1), further suggesting intensified intraspecific competition among plants. Therefore, ephemeral plants usually adopt a strategy of decreasing their survival percentage, reducing intraspecific competition, and shortening their life history to cope with intense drought stress.

### 4.2 Effects of drought pretreatment on the morphological and biomass traits of *E. oxyrhinchum*

Plants respond to environmental stress by adjusting their morphological traits and biomass allocation (Freschet et al., 2018; Geleta et al., 2024). Our study demonstrated that plants exposed to drought pretreatment showed more significant morphological changes than those in the control treatment (Figure 4). Specifically, from the initial leaf expansion to the full blooming stage, the plant height of *E. oxyrhinchum* under the drought pretreatment was significantly higher than that of the control treatment. Additionally, during the initial leaf expansion and flowering stages, the number of leaves under the drought pretreatment was significantly higher than that in the control treatment. The root length of *E. oxyrhinchum* under drought pretreatment was also significantly greater than that of the control treatment during the initial leaf expansion stage. This suggests that ephemeral plants under drought pretreatment could prioritise resource allocation to their roots, allowing them to extend deeper or wider soil layers to obtain more water and nutrients and improve their adaptability. Similarly, Xu et al. (2023) found that drought stress prompted *Alhagi sparsifolia* to allocate more resources for root growth, thereby enhancing its survival and adaptability in desert environments.

The positive correlation between morphological traits and biomass has been validated extensively (Lu et al., 2014; Chen et al., 2019b). In this study, correlation analysis also revealed a positive correlation between plant height, root length, leaf area, and leaf number, and the individual biomass of *E. oxyrhinchum* (Table 4). In addition, we found that the individual and reproductive biomass of *E. oxyrhinchum* under drought pretreatment was significantly higher than that of the control treatment. This suggests that moderate drought stress did not significantly decrease the biomass of *E. oxyrhinchum* but rather promoted its growth of *E. oxyrhinchum*, demonstrating an overcompensation effect. Similarly, in agricultural practices, managers often apply mild short-term drought stress to specific crop root zones, inducing a stress response through alternating partial root-zone irrigation, thereby promoting biomass accumulation and improving yield (Consoli et al., 2017; Fu et al., 2017; Liu et al., 2020b; Wang et al., 2024). The persistence of annual plant populations depends entirely on seed production (Lan and Zhang, 2008). In this study, drought pretreatment significantly increased the reproductive output of *E. oxyrhinchum*, which is likely related to the accelerated transition of ephemeral plants from vegetative to reproductive growth under drought stress. In the early spring in the Gurbantunggut Desert, soil moisture undergoes a gradual change from high to low, indicating that drought stress continues to intensify (Huang et al., 2015b; Chen et al., 2019d; Lu et al., 2022). To ensure population persistence, ephemeral plants must complete their reproductive output before the onset of extreme drought or hot summers (Mu et al., 2021; Lu et al., 2022). Therefore, the overcompensation effect in *E. oxyrhynchum* under drought stress is an important strategy for population persistence and community stability in the Gurbantunggut Desert.

**TABLE 4:** The correlation analysis between individual biomass and morphological traits of *Erodium oxyrhinchum* under different drought treatments.

Index	Treatment	Plant height	Root length	Number of leaves	Leaf area
Individual biomass	CK	0.912***	0.856***	0.615***	0.805***
	DP	0.929***	0.844***	0.71***	0.864***

Note: Control treatment, CK; drought pretreatment, DP; *p < 0.05, **p < 0.01, ***p < 0.001.

### 4.3 Effects of drought pretreatment on the C, N, and P stoichiometric characteristics of *E. oxyrhinchum*

C, N, and P are essential nutrients that play critical roles in plant growth and development, influencing key physiological and ecological processes such as photosynthesis, transpiration, and reproductive growth (Zhang et al., 2021; Costa et al., 2024). Our study revealed that the leaf C concentration under drought pretreatment was significantly higher than that of the control treatment during the initial and full leaf expansion stages. This suggests that drought pretreatment increases C accumulation in the early stages of growth, which is likely because plants adjust their energy storage structure under drought conditions to maintain survival and growth. A study on tropical plant seedlings found that plants allocate limited carbon resources to nonstructural carbohydrates under drought stress, which is beneficial for maintaining normal metabolic requirements and rapid recovery after drought stress (O’Brien et al., 2014). Additionally, the coefficient of variation for leaf C concentration under the control and drought treatments was less than 5%. This may be because carbon is a fundamental element of plant structure, and the C concentration in leaves generally remains relatively stable. Our study showed that the leaf N concentration of *E. oxyrhinchum* in the control and drought treatments exhibited a trend of first increased and then decreased. This may be because the growth rate of the leaves reached its maximum and required higher nitrogen absorption to synthesise proteins before the flowering stage. However, as plants grow, more N is allocated to the reproductive organs, resulting in a relative decrease in the leaf N concentration. In addition, the leaf P concentration under drought pretreatment was significantly higher than that of the control treatment. This may be due to the enhanced resistance and overcompensation effects in plants exposed to drought stress, which require more phosphorus for rRNA synthesis to support plant growth (Elser et al., 2010).

The leaf N:P ratio can be used as an indicator to assess whether plant productivity is limited by nutrients (Yuan et al., 2011; Wang et al., 2014; Lu et al., 2023). Generally, it is accepted that when N:P < 14, plant growth is more limited by nitrogen, when 14 < N:P < 16, plant growth is simultaneously limited by nitrogen and phosphorus, and when N:P > 16, phosphorus becomes the primary limiting factor for plant growth (Güsewell and Koerselman, 2002; Sardans et al., 2012; Yang et al., 2018). Our study found that leaf N:P under drought pretreatment was less than 14 during the cotyledon stage, 14 < N:P < 16 during the initial leaf expansion stage, and N:P > 16 from the full leaf expansion stage to the full fruiting stage (Figure 6F). Similarly, the leaf N:P in the control treatment was greater than 16 from the leaf expansion stage to the full fruiting stage. These findings indicated that the growth of *E. oxyrhinchum* during the cotyledon stage was primarily limited by N. After the initial leaf expansion stage, N and P limit plant growth simultaneously. The effect of P on plant growth gradually increased during the full leaf expansion, flowering, and fruiting stages. Therefore, it is necessary to optimise ecological restoration measures based on the nutritional needs of plants and improve the effectiveness of restoration.

## 5 Conclusion

Plants are often exposed to various degrees of drought under natural conditions, which are likely to affect their growth and development. Our study revealed that *E. oxyrhinchum* exposed to drought stress significantly accelerated the transition of phenological stages and shortened its life history. Meanwhile, the survival percentage of *E. oxyrhinchum* under drought pretreatment significantly decreased but showed overcompensation effects on morphological traits and biomass accumulation. This indicates that ephemeral desert plants exhibit a trade-off between survival and growth when subjected to drought stress. In addition, from the perspective of stoichiometric characteristics, *E. oxyrhinchum* subjected to drought pretreatment required more phosphorus to enhance its resistance to severe drought in the Gurbantunggut desert. Therefore, this study provides novel insights into the restoration of desert ecosystems based on plant nutritional requirements in the context of climate change.

## Data Availability

The original contributions presented in the study are included in the article/Supplementary Material, further inquiries can be directed to the corresponding author.
