# Supplementary material for: Effects of drought pretreatment on the morphology traits, biomass, and stoichiometric characteristics of the desert ephemeral plant
Source: Front Genet. 2025 Mar 20;16:1534894. doi: 10.3389/fgene.2025.1534894 (PMC11965633; doi:10.3389/fgene.2025.1534894)
Supplement: Supplementary file 1 [file Supplementaryfile1.docx]

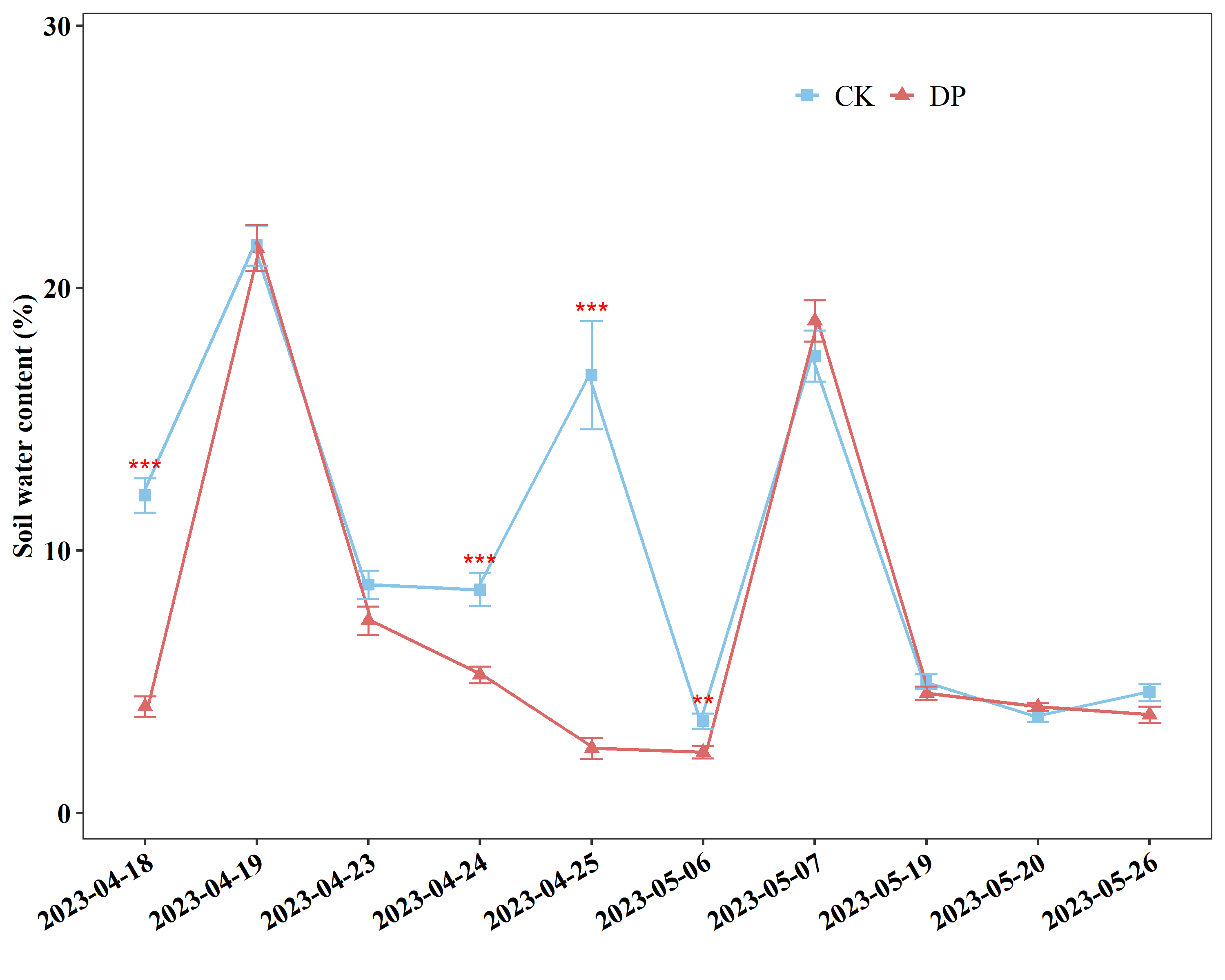


**Supplementary Figure 1.** Soil moisture content

**Note:** Control treatment, CK; Drought pretreatment, DP; * *p* < 0.05，** *p* < 0.01，*** *p* < 0.001.
